# FZD1/KLF10-hsa-miR-4762-5p/miR-224-3p-circular RNAs axis as prognostic biomarkers and therapeutic targets for glioblastoma: a comprehensive report

**DOI:** 10.1186/s12920-023-01450-w

**Published:** 2023-02-08

**Authors:** Fang Jia, Lixia Zhang, Zhengye Jiang, Guowei Tan, Zhanxiang Wang

**Affiliations:** 1 Neurosurgery Department, The First Affiliated Hospital of Xiamen University, School of Medicine, Xiamen University, 55 Zhenhai Road, Siming District, Xiamen, 361001 Fujian China; 2 Rehabilitation Department, Inner Mongolia Medical University, Hohhot, Inner Mongolia China

**Keywords:** Glioblastoma, ceRNA network, DEGs, Prognostic biomarker

## Abstract

**Background:**

The circular RNA (circRNA) plays a vital role in the pathogenesis of tumors as a competitive endogenous RNA (ceRNA). Given the high aggressiveness and fatality rate of glioblastoma (GBM) as well as poor prognosis, it is necessary to construct a circRNA-related ceRNA network for further studies on the mechanism of GBM and identify possible biomarkers as well as therapeutic drugs.

**Methods:**

Three datasets from the gene expression omnibus (GEO) database were downloaded to distinguish differential circRNAs, microRNAs, and messenger RNAs respectively in GBM. With the help of GEPIA2, circBank, CSCD, TargetScan, miRDB, and miRTarBase databases, we established a circRNAs-related ceRNA network in GBM. Functional enrichments were employed to profile the most relevant mRNAs to indirectly clarify the mechanisms of the ceRNA network. Based on the expression profile data and survival information of GBM patients from the GEO and the cancer genome atlas (TCGA) databases, we performed survival analysis to select prognostic mRNAs and constructed a novel circRNA-miRNA-mRNA central regulatory subnetwork. The DGIdb database was used to find potential drug–gene interactions.

**Results:**

The datasets obtained from the GEO and TCGA databases were analyzed, and 504 differentially expressed mRNAs (DEmRNAs), 71 differentially expressed microRNAs (DEmiRNAs), and 270 differentially expressed circRNAs (DEcircRNAs) were screened out. The novel ceRNA regulatory network included 22 circRNAs, 11 miRNAs, and 15 mRNAs. *FZD1* and *KLF10* were significantly correlated with the overall survival rate of patients with GBM (*P* < 0.05). The final survival subnetwork contained six circRNAs, two miRNAs, and two mRNAs. Two small-molecule compounds and one antibody could be used as therapeutic drugs for GBM. Interestingly, the Wnt signaling pathway appeared in both KEGG and GO functional terms.

**Conclusions:**

Results of this study demonstrate that *FZD1* and *KLF10* may exert regulatory functions in GBM, and the ceRNA-mediated network could be a therapeutic strategy for GBM.

**Supplementary Information:**

The online version contains supplementary material available at 10.1186/s12920-023-01450-w.

## Background

Glioblastoma is the most frequent primary malignant brain tumor in adults and is often rapidly fatal [1]. The associated complications impose a substantial economic burden and medical strain around the world [2]. Despite advances in current treatments, their impact on GBM remains limited, possibly due to an insufficient understanding of its pathogenesis. Hence, more studies must be conducted to investigate the mechanisms of GBM and to identify novel biomarkers for predicting prognosis and treatment outcomes.

Using high-throughput sequencing techniques, several noncoding RNA (ncRNAs) were found to be correlated with dysregulated gene expression and biological process imbalances in GBM [3].

CircRNAs are unaffected by exonucleases (more stable than linear RNAs) and typically exhibit tissue or developmental stage-specific expression, implying their biological function in various diseases, including GBM [4]. In line with this, the competing endogenous RNA (ceRNA) hypothesis mentions that circRNAs act as miRNA sponges in cells to bind to them and affect the corresponding target genes [5]. For instance, *circNT5E* controls various pathological processes such as cell proliferation, metastasis, and invasion, and is rich in *miR-422a* binding sites. CircNT5E can inhibit *miR-422a* activity by binding, showing tumor suppressor-like characteristics in GBM [6]. Besides, the functions of circRNAs (including *circCCDC66*, *circSMARCA5*, *circMTO1*) have been gradually discovered, especially their ability to bind miRNAs in cervical cancer, hepatocellular carcinoma, and glioma pathology [7–10]. Therefore, we hypothesize that the expression of miRNA is negatively correlated with the expression of circRNA and mRNA. The importance of ceRNA in tumorigenesis is self-evident, but the roles of the circRNA–mRNA–miRNA ceRNA network in GBM remain unclear.

This research involved the comprehensive analysis of multiple databases. Furthermore, this research analyzed the effect of differentially expressed genes (DEGs) on GBM overall survival, constructed a prognosis-related subnetwork to strengthen the understanding of the pathogenesis of GBM, and proposed several drugs that might be potential therapeutic agents for GBM.

## Materials and methods

### Data obtained

We downloaded expression profiles of the GSE165926, GSE103229, and GSE90886 datasets from the GEO database web resource (https://www.ncbi.nlm.nih.gov/geo/). The GSE165926 dataset contained circRNA data from 4 normal brains and 12 GBM tissues [11]. The GSE103229 dataset, which comprised microRNA expression profiling, included 5 normal brain tissues and 5 GBM tissues. The GSE90886 dataset included mRNA expression profiles of 9 normal tissues and 9 GBM tissues [12]. To verify the preliminary findings, the serial expression profiles of glioblastoma were downloaded from the TCGA database (http://cancergenome.nih.gov/), including data from 5 non-tumor brains and 169 GBM samples for survival analysis and collection of clinical information on cases.

### Identification of differentially expressed genes (DEGs)

The annotation documents of the corresponding platforms were used for gene expression profiling. The average value of gene expression was used when one gene corresponded to multiple probes. The “LIMMA” package in R software (version 4.1.1) was employed to identify differentially expressed miRNAs, circRNAs, and mRNAs between glioblastomas and non-tumor brain tissues in both GEO datasets and TCGA datasets, with thresholds respectively set at |log2 fold change (FC)|≥ 2.0, 2.0, 1.0; *P* < 0.05 [13]. Next, the differentially expressed genes (DEGs) from the GEO and TCGA datasets were divided into upregulated and downregulated groups for further analysis. Venn diagrams were drawn using the “Venn” package of R software (version 4.1.1).

### ceRNA network establishment

Firstly, we converted the names of DEcircRNAs to mature circRNA names using the circBASE database web source (http://www.circbase.org/). Then the CSCD database (web source: http://gb.whu.edu.cn/CSCD/) was employed to find the miRNAs response elements (MREs) by entering circRNAs names. Only the interactions supported by solid evidence (reporter assays, western blots, and qPCR) were included in the prediction to enhance the reliability of the results. To further investigate the role of mRNAs in GBM, we used the TargetScan (http://www.targetscan.org/), miRTarBase (http://mirtarbase.cuhk.edu.cn/php/index.php), and miRDB (https://www.mirbase.org/) databases to find miRNAs target genes [14]. We compared the target genes and DEmRNAs, retaining only data for intersecting mRNAs and corresponding miRNAs for further study. According to the prediction and evaluation above, a visualized circRNAs-miRNAs-mRNAs network was constructed using Cytoscape software (version 3.8.2,

http://www.cytoscape.org/). Statistical significance was only determined for terms with a *P* < 0.05.

### Functional enrichment analysis

ClusterProfiler is an R package that provides three methods, enrichGO, enrichKEGG, and groupGO, for enrichment analysis and classification [15]. We used it to perform the Kyoto Encyclopedia of Genes and Genome (KEGG) and Gene Ontology (GO) pathway enrichment analyses of DEmRNAs in the circRNAs-miRNAs-mRNAs network. *P* < 0.05 was set as the criterion for statistical significance, and the terms were visualized via the “ggplot2” package in R.

### Construction of a protein–protein interaction (PPI) network

A PPI network was established by the Search Tool for the Retrieval of Interacting Gene (STRING, https://string-db.org/), an online database of billions of interactions for thousands of organisms [16] and visualized via Cytoscape software (version 3.8.2). The list of DEmRNAs was first mapped to the STRING locus, and their interactions were evaluated. In addition, the genes were selected based on the PPIs comprehensive score > 0.9 and the degree of close correlation with other genes adjusted to ≥ 10 [16]. The Molecular Complex Assay (MCODE) based on a weighted algorithm was then used to identify highly interacting gene modules in the PPI network, and the standard parameters were set by default, except k-core = 7 [17].

### Survival analysis and prognostic network construction

To determine critical genes associated with the survival of GBM patients, the prognostic values of the DEmRNAs were estimated with the clinical information acquired from TCGA. The “survival” package in R software was employed to find associations between mRNAs and survival status data [18]. FDR < 0.05 was statistically significant. The association of *FZD1* (Frizzled class receptor 1) and *KLF10* (Krüppel-like factor 10) with immune infiltration in GBM was analyzed in an online database: TIMER (web source: https://cistrome.shinyapps.io/timer/), which can investigate immune cell infiltration levels using data from TCGA. We used Cytoscape software (version 3.8.3) to construct the prognostic circRNA-miRNA-mRNA ceRNA subnetwork.

### Drug–gene interaction

Through the interaction, DEmRNAs were paired with existing compounds to discover potential therapeutic agents for GBM. The drug–gene interaction database (DGIdb, web source: https://dgidb.org/) provides an interface for searching gene lists based on drug–gene interaction profiles and potentially "druggable" genes [19]. DEmRNAs were uploaded into DGIdb to match with the existing drugs.

### Meta-analysis

To validate our findings, the eligible published studies were searched by the following databases: Chinese CNKI, Cochrane Library, Web of Science, and PubMed. Citations from the inclusive studies were also selected for additional relevant studies. We used “*FZD1*” or “*fzd1*” or “Frizzled class receptor 1” AND “neoplasms” or “cancer” or “tumor” or “carcinoma”, “*KLF10*” or “*klf10*” or “Krüppel-like factor 10” AND “neoplasms” or “cancer” or “tumor” or “carcinoma” as the search terms for appropriate identification. The exclusion and inclusion criteria are shown in Additional file 1: Table 1. We extracted the following information: 1st author, country, date of publication, patients’ number, cancer type, sample assay approach, threshold value of *FZD1* and *KLF10* expression level, 95% CI and HR of overall survival (OR). We recorded 95% CI and HR directly if they were available in the studies. If not, we analyzed and abstracted the data from Kaplan–Meier (K–M) curves of OS based on the approach described by a previous research [20]. Two researchers evaluated the data independently, a third investigator decided whether to incorporate the study. The Newcastle–Ottawa Scale (NOS) was used to evaluate the quality of the enrolled studies [21].

**Table 1:** The GO function enrichment analysis terminologies for mRNAs in this ceRNA network

ID	Description	*P* value	Count	Gene names
GO:0001503	Ossification	0.000251	4	FZD1/SOX11/DDX21/KLF10
GO:0001649	Osteoblast differentiation	0.000737	3	FZD1/SOX11/DDX21
GO:1901016	Regulation of potassium ion transmembrane transporter activity	0.001324	2	NETO2/KCNE4
GO:1901379	Regulation of potassium ion transmembrane transport	0.002562	2	NETO2/KCNE4
GO:1990909	Wnt signalosome	0.009171	1	FZD1
GO:0000242	Pericentriolar material	0.016753	1	DYRK3
GO:0090543	Flemming body	0.02653	1	CEP55
GO:0016607	Nuclear speck	0.038815	2	PLCB1/DYRK3
GO:0032154	Cleavage furrow	0.039916	1	CEP55
GO:0008081	Phosphoric diester hydrolase activity	0.00234	2	PLCB1/ENPP2
GO:0004620	Phospholipase activity	0.003177	2	PLCB1/ENPP2
GO:0016298	Lipase activity	0.00484	2	PLCB1/ENPP2
GO:0001228	DNA-binding transcription activator activity, RNA polymerase II-specific	0.005337	3	HOXA10/SOX11/KLF10

GO, Gene ontology, mRNAs, messenger RNA, ceRNA, competitive endogenous RNA

### Statistics

Statistical analyses were performed with the R software (version 4.1.1) and the above packages. Meta-analysis was conducted using the SPSS software. Chi-square-based I2 tests and Cochran’s Q were conducted to test for heterogeneity. I^2^ < 50%, *P* > 0.10 indicates no significant heterogeneity across studies. Subgroup analyses were applied to investigate the sources of heterogeneity. The 95% CI and HR in each study were integrated and plotted as forest plots to assess the impact of *FZD1* and *KLF10* expressions on OS in cancers. Sensitivity analysis was used to examine the reliability of the results. Two-tailed *P* values < 0.05 were considered statistically significant.

## Results

### DEGs detection

**Fig. 1:**
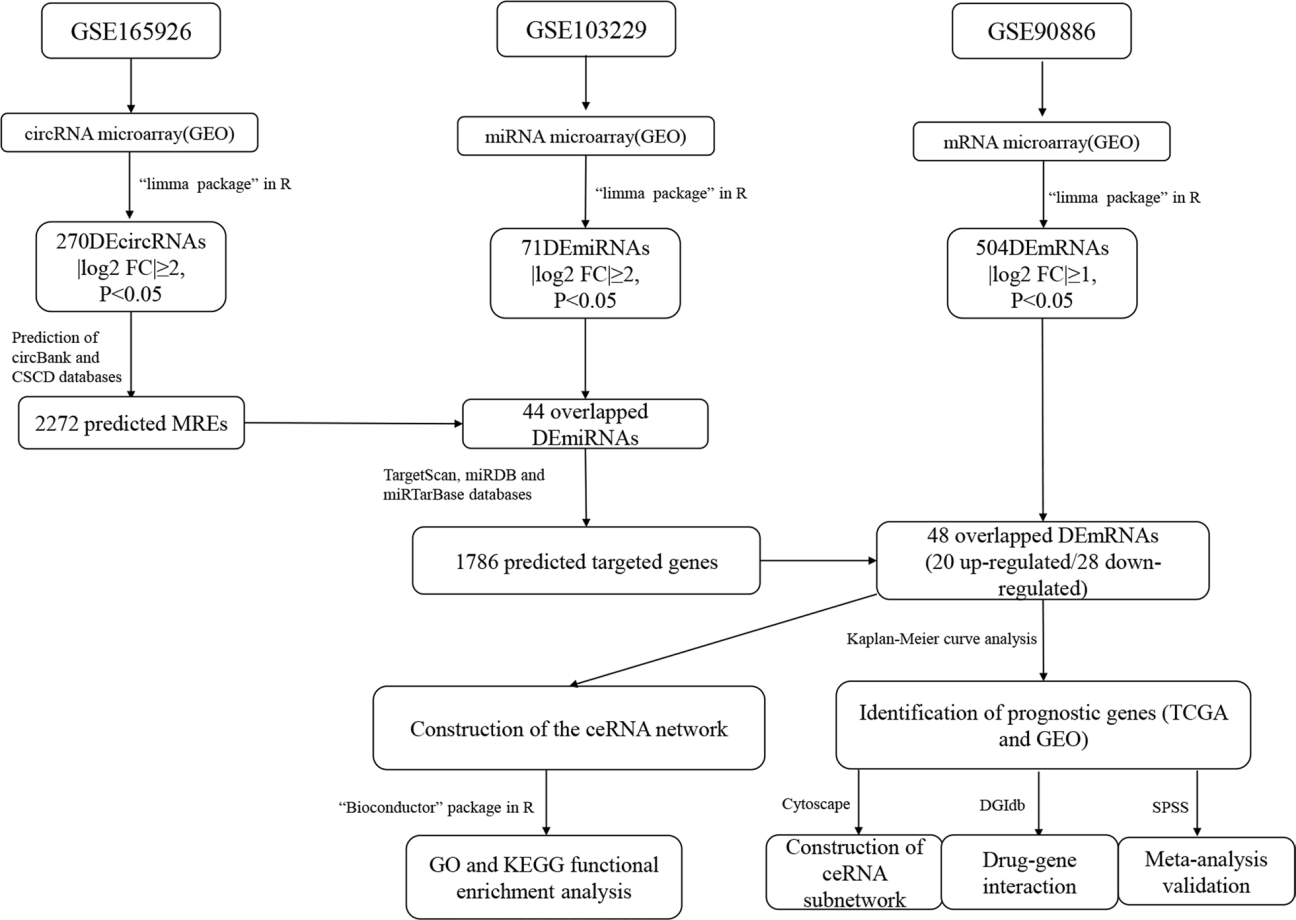
The basic framework of this analysis

**Fig. 2:**
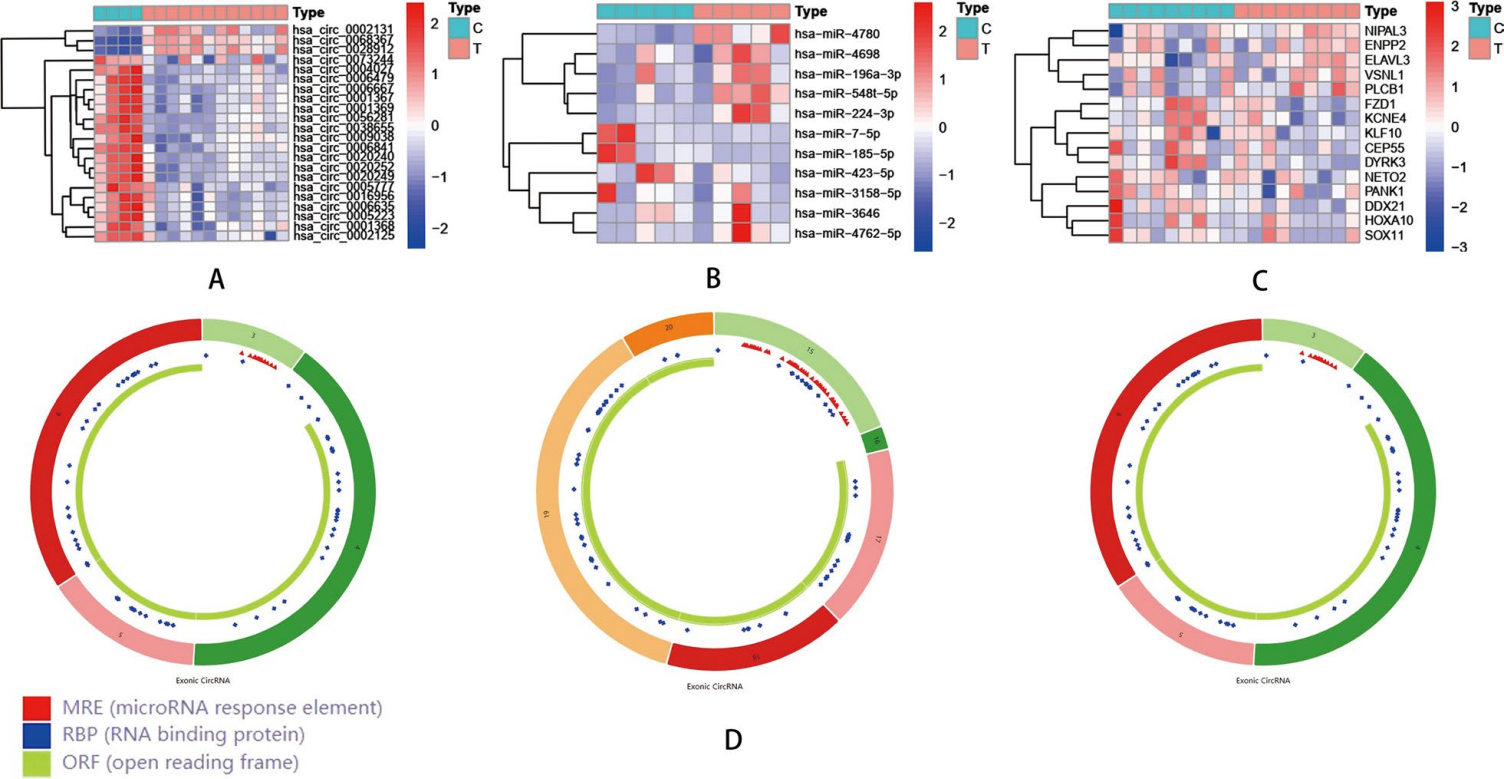
Expression profile heatmaps for DEGs were screened from three datasets in the ceRNA network. **A** represents the heatmap of circRNAs, **B** represents the miRNAs' heatmap, and **C** represents the mRNAs' heatmap. **D** Structure diagrams of circRNAs; the different colors, shapes of the outer and inner loops represent the different exons and locations of MRE, RBP, and OR

Figure 1 displays the basic flow chart of this study. Firstly, 270 DEcircRNAs, including 183 down-regulated genes and 87 up-regulated genes, were identified from normal controls and GBM samples in the GSE165926 dataset by the “limma” package of R software, by setting the cutoff value as |log_2_ fold change (FC)|≥ 2, *P* < 0.05; Similarly, 71 DEmiRNAs were screened in the GSE103229 dataset, including 34 down-regulated and 37 up-regulated genes; the criterion was set as |log_2_ FC|≥ 2, *P* < 0.05. Meanwhile, the GBM samples and normal control group in the GSE90886 dataset were analyzed, and 504 DEmRNAs were obtained, including 242 down-regulated genes and 262 up-regulated genes, with thresholds set at |log_2_ FC|≥ 1, *P* < 0.05. We showed the heatmaps of some DEGs and several circRNAs in Fig. 2A–C. The structures of several DEcircRNAs are displayed in Fig. 2D.

### Construction of the ceRNA network

**Fig. 3:**
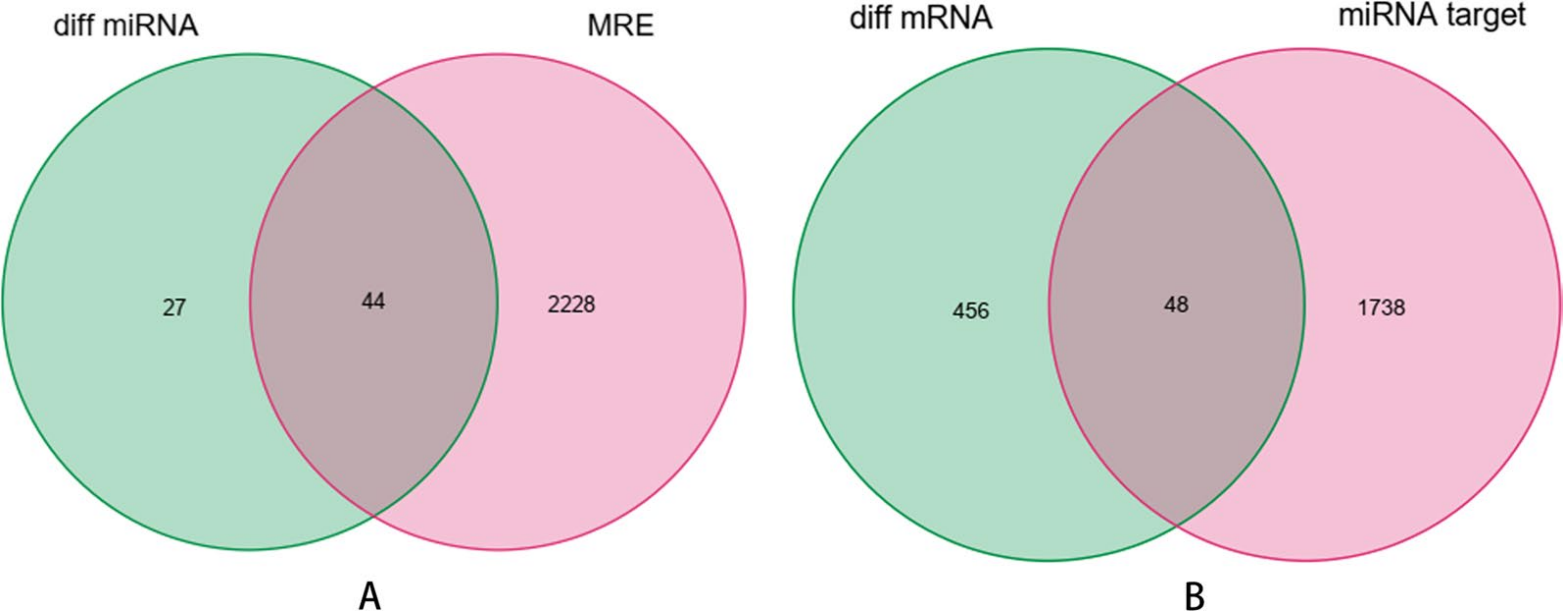
Venn diagram showing the 44 overlapped diff miRNAs identified with the intersection of GSE103229 and GSE165926 datasets, 48 target diff mRNAs identified with the intersection of two gene sets

**Fig. 4:**
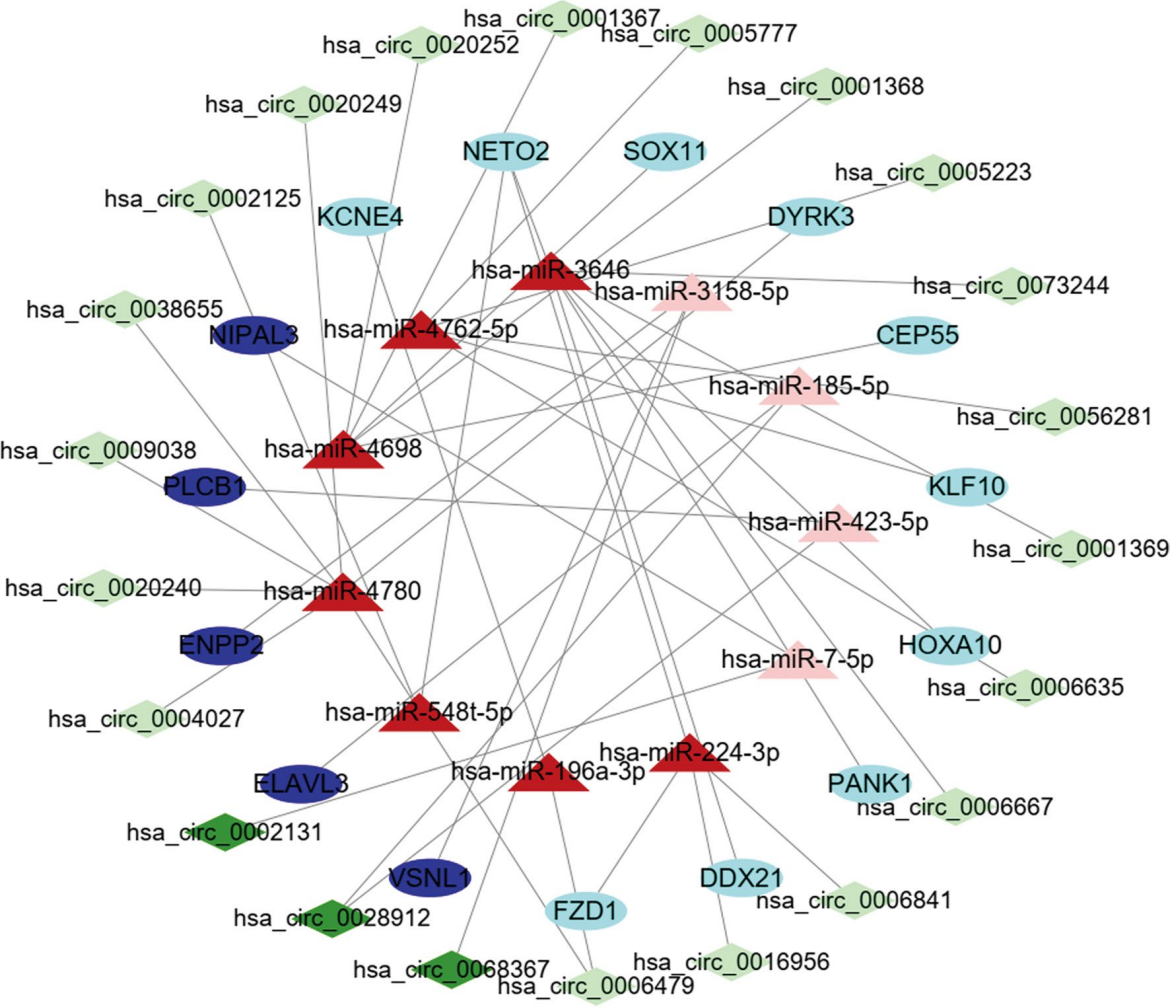
The ceRNA network about circRNAs-miRNAs-mRNAs. Light red triangular nodes represent the downregulated miRNAs, dark red triangular nodes represent the upregulated miRNAs; Light green diamonds represent the downregulated; dark green diamonds represent the upregulated circRNAs; Light blue elliptical nodes represent the downregulated, dark blue elliptical nodes represent the upregulated mRNAs

**Fig. 5:**
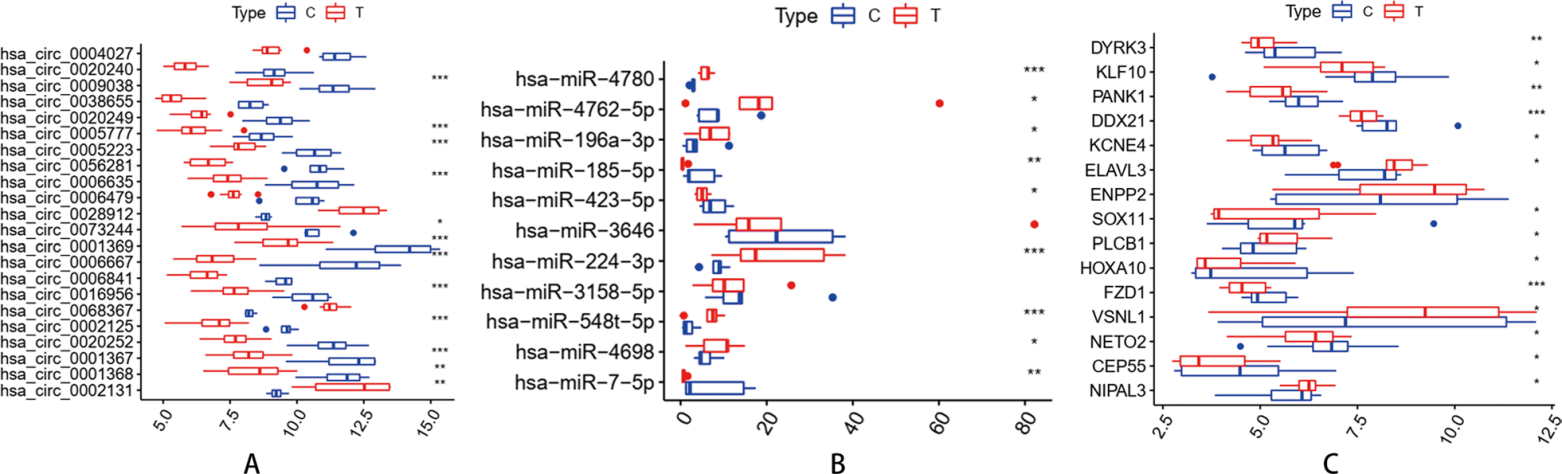
Expression boxplots of mRNAs, miRNAs circRNAs in the ceRNA network. **A** The boxplot of circRNAs; **B** the boxplot of miRNAs; **C** the boxplot of mRNAs. “*” represents *P* value < 0.05, “**”represents *P* value < 0.01, “***” represents *P* value < 0.001

To further ensure the reliability of differentially expressed RNAs and the ceRNA network in GBM, we only retained the overlapping parts from the three datasets, we selected circRNAs-miRNAs interaction pairs and miRNAs-mRNAs interaction pairs from TargetScan, miRDB, and miRTarBase databases to construct a circRNAs-miRNAs-mRNAs ceRNA network. Results showed that 44 of 71DEmiRNAs in the GSE103229 dataset were the same as the integrated miRNAs in the GSE165926 dataset, and 48 of 504 DEmRNAs in the GSE90886 dataset corresponded with the miRNA-targeted genes likewise (Fig. 3). A total of 11 DEcircRNAs and 15 DEmRNAs constituted 17 interacting pairs. In addition, 22 DEcircRNAs and 11 DEmiRNAs formed 24 interaction pairs. Afterwards, 22 DEcircRNAs, 15 DEmRNAs, and 11 DEmiRNAs were used to establish a ceRNA network using Cytoscape software (version 3.8.2) for visualization (Fig. 4). We also drew the boxplots of DEcircRNAs, DEmiRNAs, and DEmRNAs in the ceRNA network, respectively (Fig. 5A–C).

### Function and pathway enrichment analysis

**Fig. 6:**
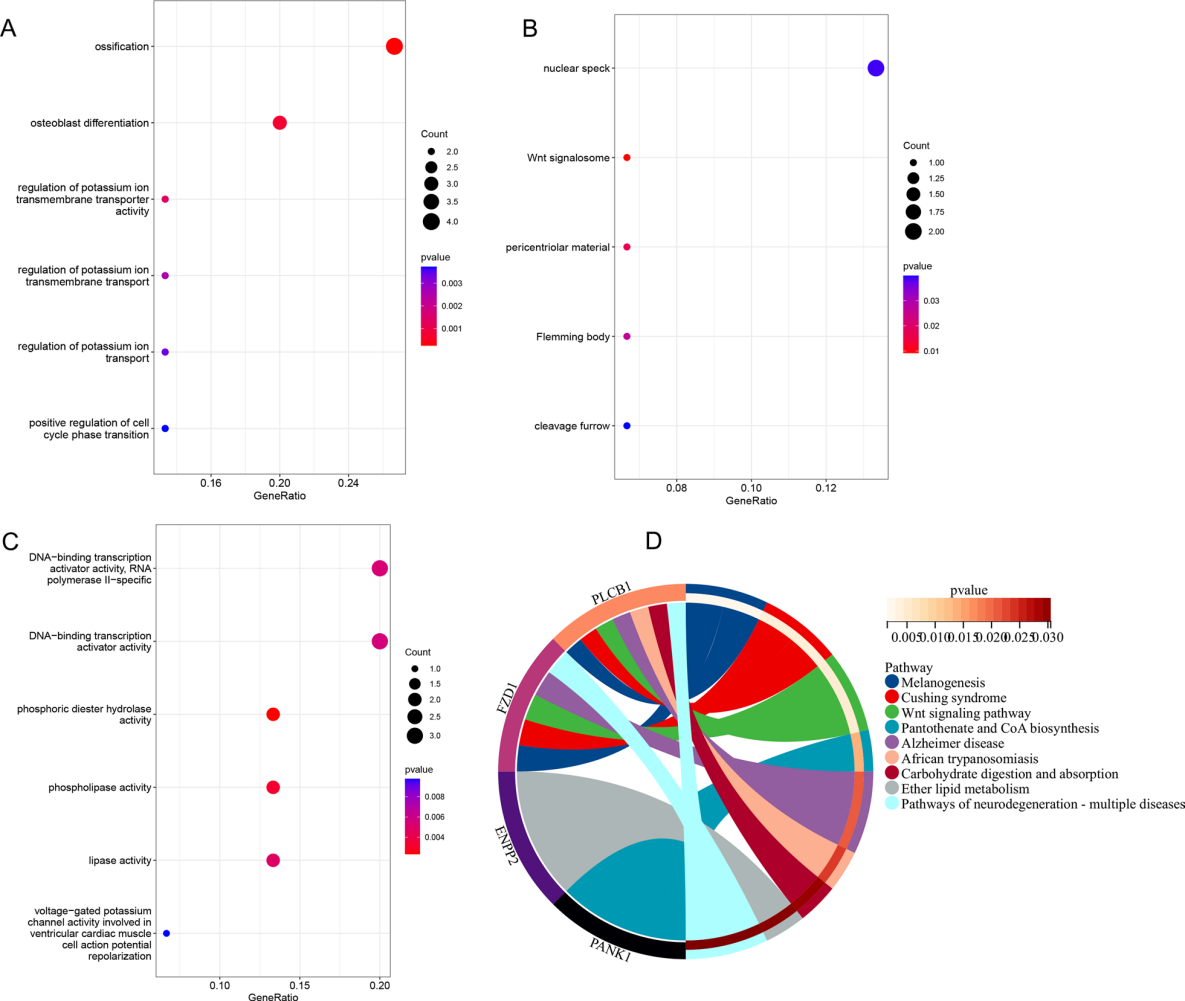
GO enrichment analysis of mRNAs in the ceRNA network and KEGG pathway analysis of mRNAs in the ceRNA network. **A** Represents the “biological process”. **B** Represents the “cellular component”. **C** Represents the “molecular function”. **D** Represents the “KEGG Enrichment Analysis Circle Plot”

We performed the KEGG pathway and GO functional enrichment analysis using DEmRNAs in the ceRNA network. GO results revealed that DEmRNAs were more related to the ossification (BP), Wnt signalosome (CC), and phosphoric diester hydrolase activity (MF) (Fig. 6A–C; Table 1), respectively. KEGG analysis revealed 19 statistically significant enriched pathways. The top-3 most enriched pathways were: Melanogenesis, Cushing syndrome, Wnt signaling pathway (Fig. 6D; Table 2).

**Table 2:** The KEGG signaling pathway analysis terms of mRNAs in the ceRNA network

ID	Description	P value	Count	Gene names
hsa04916	Melanogenesis	0.001497	2	FZD1/PLCB1
hsa04934	Cushing syndrome	0.003491	2	FZD1/PLCB1
hsa04310	Wnt signaling pathway	0.004042	2	FZD1/PLCB1
hsa00770	Pantothenate and CoA biosynthesis	0.012877	1	PANK1
hsa05010	Alzheimer disease	0.020311	2	FZD1/PLCB1
hsa05143	African trypanosomiasis	0.022599	1	PLCB1
hsa04973	Carbohydrate digestion and absorption	0.028636	1	PLCB1
hsa00565	Ether lipid metabolism	0.029839	1	ENPP2
hsa05022	Pathways of neurodegeneration—multiple diseases	0.030506	2	FZD1/PLCB1
hsa04961	Endocrine and other factor-regulated calcium reabsorption	0.032244	1	PLCB1
hsa04730	Long-term depression	0.036439	1	PLCB1
hsa05217	Basal cell carcinoma	0.038233	1	FZD1
hsa04929	GnRH secretion	0.03883	1	PLCB1
hsa04927	Cortisol synthesis and secretion	0.039427	1	PLCB1
hsa04720	Long-term potentiation	0.04062	1	PLCB1
hsa04924	Renin secretion	0.041812	1	PLCB1
hsa00562	Inositol phosphate metabolism	0.044193	1	PLCB1
hsa04918	Thyroid hormone synthesis	0.045381	1	PLCB1
hsa04971	Gastric acid secretion	0.045975	1	PLCB1

KEGG, Kyoto encyclopedia of genes and genome, mRNAs, messenger RNA, ceRNA, competitive endogenous RNA

### PPI network and module analysis

**Fig. 7:**
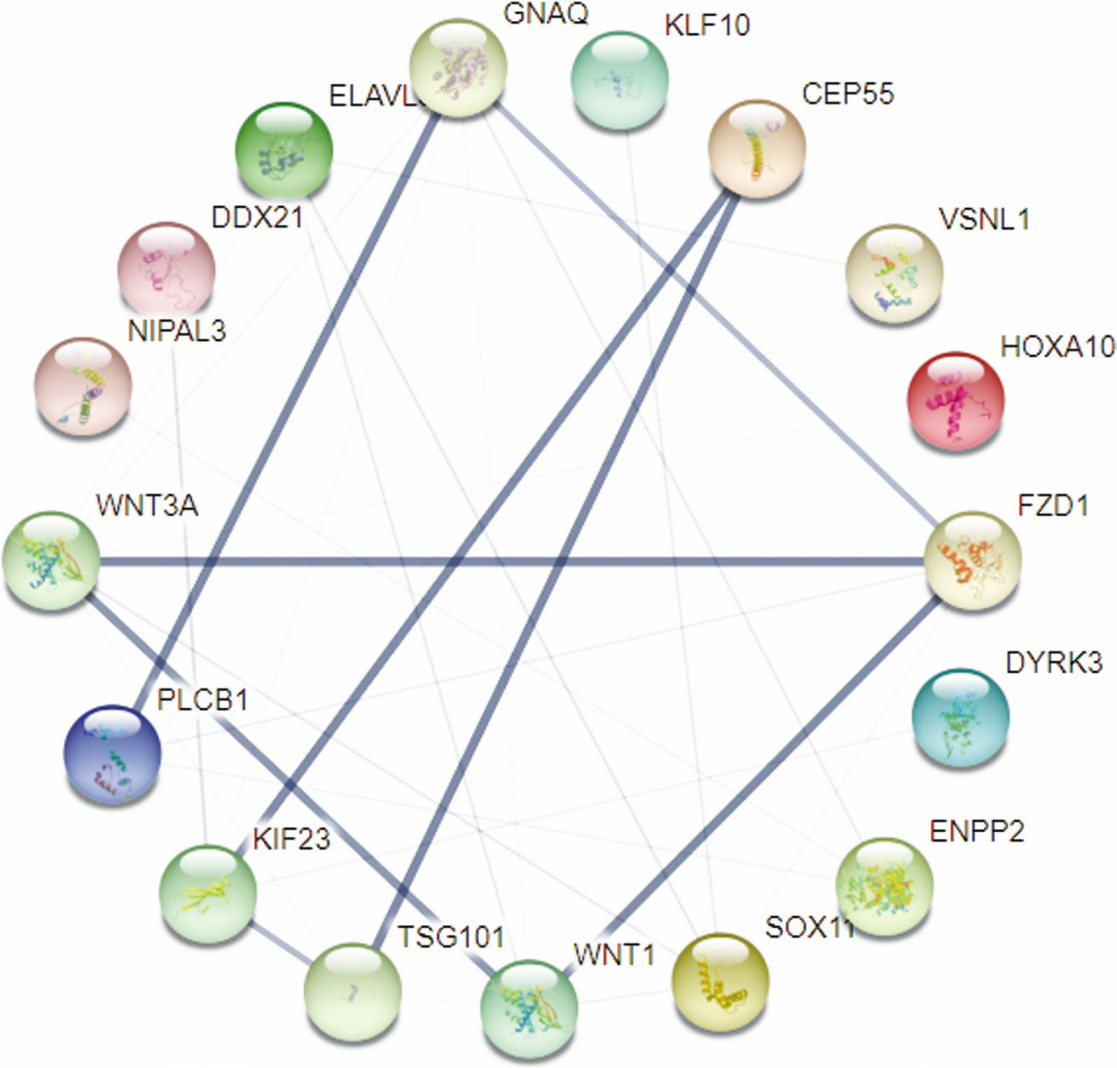
Construction of a PPI network using mRNAs in the ceRNA network. Line thickness represents the strength of data support from the side

We investigated the relationship between DEmRNAs in the network through the online tool STRING database. Then we use the plugin MCODE to select a more densely connected module with 30 edges, 17 nodes and a score of > 0.900(the highest confidence level) in this PPI network. (Fig. 7).

### DEmRNAs associated with the overall survival of GBM patients

**Fig. 8:**
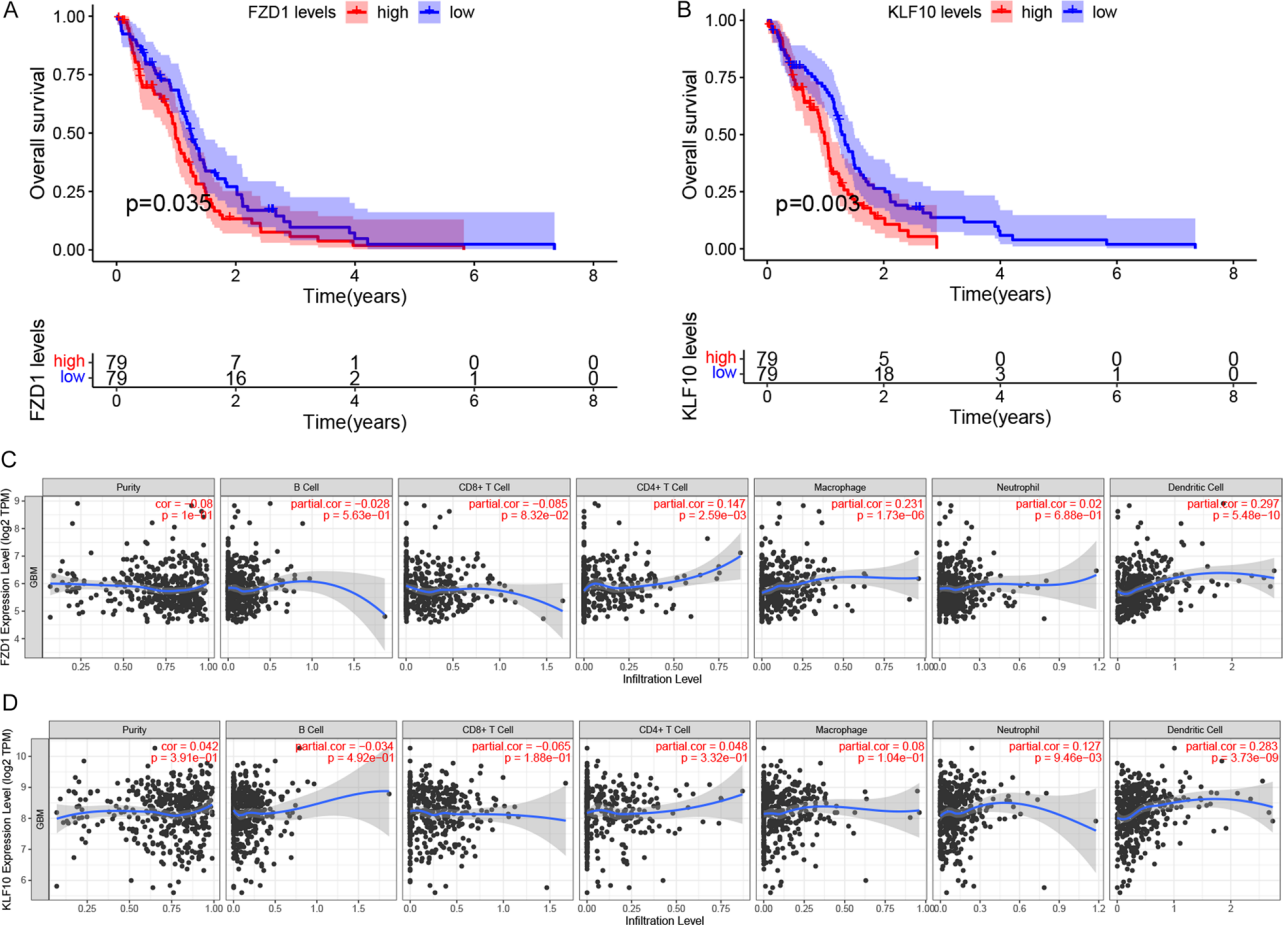
**A**, **B** Association between overall survival and mRNAs in this network analyzed via K–M survival curves. Two mRNAs were significantly correlated with the overall survival of GBM patients (FDR < 0.05), they were *FZD1* and *KLF10*. **C**, **D** The correlation between *FZD1*, *KLF10* and immune infiltration of GBM was studied using the TIMER database

**Fig. 9:**
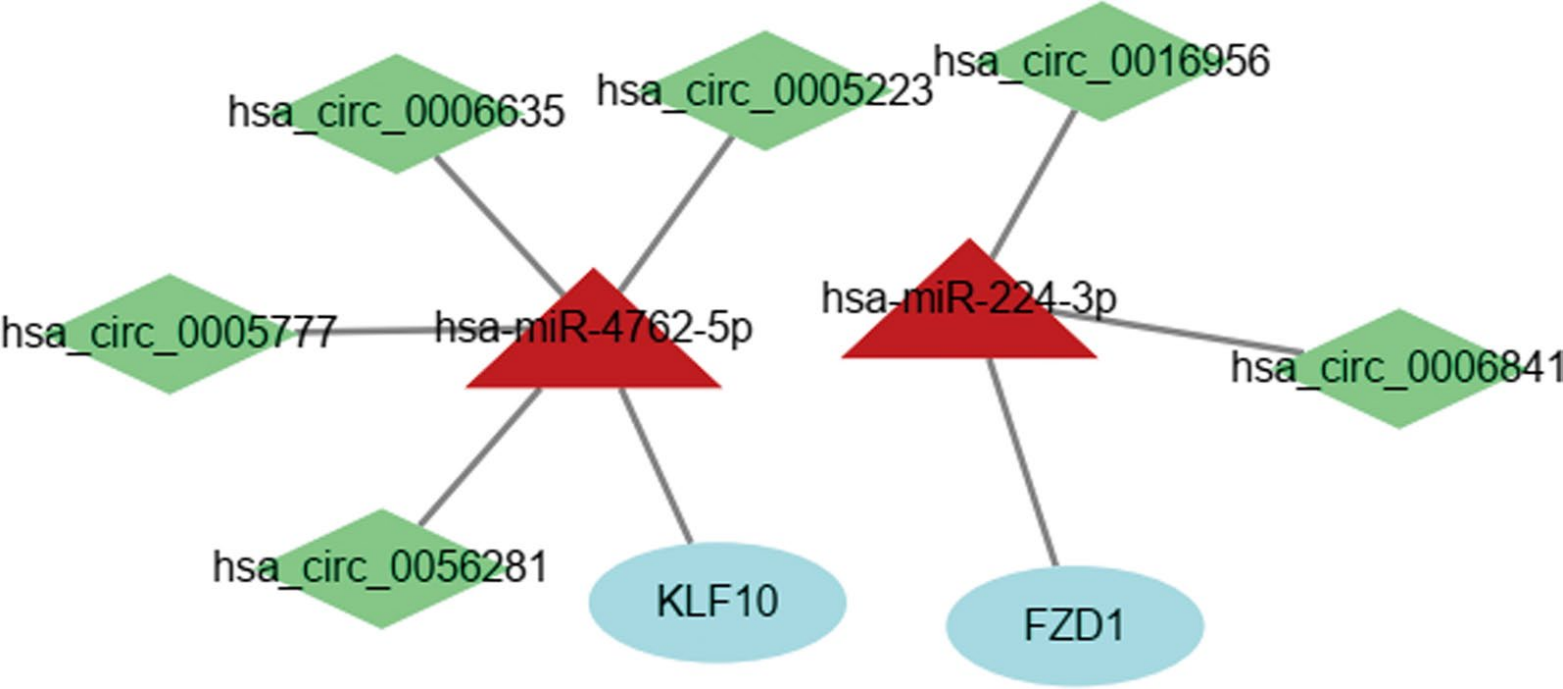
The prognostic subnetwork of circRNA-miRNA-mRNA. Light green diamonds represent downregulated circRNAs, light blue elliptical nodes represent downregulated mRNAs, and dark red triangular nodes represent upregulated miRNAs

To investigate the prognostic ability of DEmRNAs in GBM, 15 DEmRNAs in the circRNA-associated ceRNA networks were analyzed using the "survival" package of the R software (version 4.1.1). The results indicated that 2 out of 15 DEmRNAs were significantly correlated with the overall survival in GBM (FDR < 0.05). The two DEmRNAs were *FZD1* and *KLF10* and were inversely correlated with overall survival, as is shown in Fig. 8A, B. To better understand the roles of two hub genes in the GBM immune microenvironment, we analyzed the association between *FZD1*, *KLF10*, and several common immune cell types in the TIMER database. As shown in Fig. 8C, D, *FZD1* was positively correlated with the infiltrations of CD4 + T cells, macrophages, neutrophils, and dendritic cells in GBM. Strong positive correlations existed between *KLF10* expression and the infiltrations of neutrophils and dendritic cells in GBM. Finally, the related ceRNA subnetwork was also constructed using the Cytoscape software (version 3.8.2), as shown in Fig. 9.

### Drug–gene interaction

Drug–gene interactions analysis was performed on the 15 mRNAs in this circRNA-associated ceRNA network. According to the results, four drugs interacted with gene *FZD1* (frizzled class receptor (1), six drugs significantly interacted with *ENPP2* (ectonucleotide pyrophosphatase /phosphodiesterase (2). At the same time, *DYRK3* (dual specificity tyrosine-phosphorylation-regulated kinase (3) were associated with 11 different drugs. Of the two drugs, only three were found to have antineoplastic activities in GBM therapy and targeted *FZD1* and *ENPP2* genes, respectively. The 2D structural diagrams of these compounds are shown in Additional file 2: Figure S1.

### The relationship between FZD1 and KLF10 expression and prognosis

We performed the meta-analysis to initially validate the prognosis ability of *FZD1* and *KLF10*. Based on the above-mentioned criteria, six *FZD1*-related articles [22–27] including 972 patients were finally included and five *KLF10*-associated articles [28–32], consisting of 665 patients, were ultimately enrolled. Specific information on these studies is presented in Additional file 1: Tables 2 and 3. The results revealed that high expression of *FZD1* was significantly associated with poor OS (HR 2.55; 95% CI 1.95–3.34; *P* < 0.00001) (Additional file 2: Fig. 2A). The heterogeneity of the data was not significant (I^2^ = 39%, *P* = 0.15), therefore the fixed-effect model was applied. As presented in Additional file 2: Fig. 2B, high *KLF10* expression was significantly correlated with an inferior OS (HR 1.41; 95% CI 1.02–1.97; *P* = 0.04). There was no heterogeneity (I^2^ = 16%, *P* = 0.32), so a fix-effects model was used. Subsequent subgroup analyses were conducted by analyzing the record method, detecting approach, and cancer type to stratify patients. The results are shown in Additional file 1: Tables S4 and S5. And no significant bias was found by performing the funnel plot analysis (Additional file 2: Fig. 3A, B).

## Discussion

Glioblastoma (GBM) is one of the deadliest cancers in humans [33]. Treatment guidelines recommend surgical resection for newly diagnosed glioblastoma, followed by adjuvant radiation therapy and adjuvant chemotherapy [34]. Despite an aggressive treatment approach for GBM patients, median survival is generally less than one year from the date of diagnosis, and most patients die within two years, even in the most favorable circumstances [35]. Differential genes have been reported to play a key role in tumor pathology and thus may be potential targets for tumor therapy [36, 37]. This study investigated the pathological mechanism of GBM to determine and identify potential therapeutic agents for GBM. Previous studies have shown that circRNAs, miRNAs, and mRNAs play a significant role in the pathological formation of tumor processes [37–39], but little attention has been paid to the overall regulatory role of these genes in tumors, including GBM.

In this research, we mined three datasets from the GEO database to identify differentially expressed circRNAs, miRNAs, and mRNAs between normal brains and GBM tissue samples. The intersecting miRNAs and mRNAs were selected from these three datasets using bioinformatics techniques, and a circRNAs-miRNAs-mRNAs ceRNA network was established. The functions and enrichment pathways of mRNAs in this network were further explored by the GO and KEGG analyses. We then downloaded the clinical data of GBM from the TCGA database, analyzed the clinical information of relevant genes in this network, and identified 2 mRNAs, 6 circRNAs, and 2 miRNAs associated with the overall survival of GBM. Ultimately, the online drug database DGIdb was used to analyze the DEmRNAs in this network and screen three compounds that could be potential drugs for GBM.

GO analysis showed that mRNAs were enriched in some major regions, such as ossification, Wnt signalosome, and phosphoric diester hydrolase activity. And the KEGG results indicated that the top-3 most enriched pathways were Melanogenesis, Cushing syndrome, and Wnt signaling pathway, some of which have been reported in previous research regarding cancer. Melanogenesis plays an critical regulatory role in the differentiation, proliferation, apoptosis, and migration of GBM and melanoma [40]. Paraneoplastic Cushing's syndrome, caused by ectopic adrenocorticotropic hormone (ACTH) secretion, has been reported to be associated with neuroendocrine prostate cancer, small-cell lung cancer, and a variety of tumors [41, 42].

Interestingly, we found that the "Wnt signaling pathway" appeared in both GO and KEGG enrichment analyses. The WNT (Wingless/Integral) signaling pathway is involved in different stages of GBM. It has been recognized as a marker of a therapeutic challenge due to its critical function in normal tissue homeostasis [43]. Wnt signaling acts as an essential regulator of critical cellular events in the developing brain. More specifically, it is essential for self-renewal, differentiation, proliferation, and migration in neurons, astrocytes, oligodendrocytes, and so on [44]. Numerous studies have shown that excessive activation of Wnt signaling was associated with the malignant transformation and development of brain tumors [45–47]. Diclofenac and celecoxib inhibit tumor cell proliferation in a variety of human malignancies and are potential therapeutic drugs targeting glioblastoma cells that work by inhibiting activation of Wnt/β-catenin/Tcf signaling [48].

This constructed ceRNA network consisted of the core DEGs after being intersected from three datasets. We believe that this network contributes to further understanding of GBM pathogenesis at the genetic level. Some of these interactions have been demonstrated in previous reports. For instance, MiR-144, a tumor suppressor, inhibits cell viability, invasion and cell migration by downregulating *CEP55* and induces apoptosis and cell cycle arrest in breast cancer [49]. This discovery provided new prognostic biomarkers and therapeutic targets for the development of breast cancer diagnosis and treatment. Also, MiR-494 inhibits the expression of the oncogene *HOXA10* and thereby reduces the proliferation of oral cancer tissue cells [50]. And overexpression of circRNA_102171 has been reported to promote the occurrence and progression of papillary thyroid carcinoma (PTC) by activating the Wnt/β-catenin pathway in a CTNNBIP1-dependent manner [51]. In addition, several mRNAs in this ceRNA network have been found to have regulatory roles in GBM, such as *VSNL1*, *CEP55, HOXA10*, *ENPP2* [52–55], and so on. We believe that with the deepening of GBM basic and clinical trials, more and more gene targets and corresponding therapeutic drugs will be found in this ceRNA network.

To confirm the credibility of our analysis, we again built a PPI network with DEmRNA, where 12 mRNAs in the ceRNA network appeared in the most important modules. The 12mRNAs were: *NIPAL3*, *CEP55*, *VSNL1*, *FZD1*, *HOXA10*, *PLCB1*, *SOX11*, *ENPP2*, *ELAVL3*, *DDX-21*, *KLF10*, *DYRK3*, some of which have been found to play an important role in the pathological process of GBM. Clinical information, including overall survival, downloaded from the TCGA database was used to analyze the prognostic ability of genes in the circRNAs-associated ceRNA network. Six of 22 circRNAs, two of 11 miRNAs, and two of 15 mRNAs were associated with the patient's overall survival rate. These genes were *miR-224-3p*, *miR-4762-5p*, *circ_0016956*, *circ_0006841*, *circ_0006635*, *circ_0056281*, *circ_0005223*, *circ_0005777*, *FZD1*, *KLF10*. Among them, we focused on the downregulated mRNAs *(FZD1* and *KLF10*) significantly correlated with overall survival in GBM.

It has been reported in many cancer tissues that overexpression of *FZD1* can lead to tumor progression and drug resistance [25]. *FZD1* is the first member of the Frizzled gene family that translates seven-transmembrane proteins, which are receptors for the Wnt signaling pathway [56]. *FZD1* could also regulate the cellular response in physiological and pathological microenvironmental conditions [57]. Previous research found that the gradient effect of *FZD1* expression in the tumor microenvironment may regulate colon cancer progression and spread, providing a new therapeutic target for colon cancer patients [58]. *FZD1* appears to mediate drug resistance by modulating the Wnt/β-catenin pathway in clear cell renal cell carcinoma [25], neuroblastoma [59], pancreatic ductal adenocarcinoma [27], ovarian cancer [60], and breast cancer [61]. *KLF10* (Krüppel-like factor 10) has been established in several studies for its role as a tumor suppressor in cancer [62]. *KLF10* has the potential to be a marker of various diseases, including cardiac hypertrophy [62], diabetes [63], osteoporosis [64], immune system diseases, and colitis [65]. *KLF10* plays a significant role in many conditions and biological processes, including tumorigenesis. The expression of *KLF10* was negatively correlated with the progressive worsening of pancreatic cancer; therefore, *KLF10* may be used as a predictor of pancreatic cancer staging [28]. Moreover, *KLF10* expression can also serve as an independent prognostic marker in oral cancer patients, especially those in early-stage [30]. *KLF10* inhibits β-catenin nuclear transformation and Wnt signaling pathways in vivo, supporting its potential therapeutic target of multiple myeloma [66]. Research by Marrero, D et al. found that changes in *KLF10* and mRNA expression and variation in PSG copy number may be new molecular markers of cervical cancer [67]. The results of this study further demonstrate the potential of hub genes involved in the network in assessing the prognosis of GBM.

Glioblastoma is one of the most drug-resistant malignancies and often recurs after chemotherapy. Therefore, there is some need to explore new compounds or drugs to achieve the desired therapeutic effect. Three drugs (Niclosamide, Vantictumab against *FZD1*; Ziritaxestat against *ENPP2*) were identified as potential candidates with antitumor activity that may play a role in the treatment of GBM.

Vantictumab is a fully-humanized monoclonal antibody that binds to the frizzled (*FZD*) receptor and inhibits the classical WNT signaling pathway [68]. A combination of vantictumab and paclitaxel has shown promising clinical activity in patients with metastatic or locally advanced breast cancer who have received 2 prior routes of chemotherapy [69]. In addition, in the study of limiting the dilution of pancreatic transplantation, vantictumab could reduce the frequency of activity of initiating tumor cells in vitro, further supporting its effect on tumor stem cells [69].

Niclosamide has significant inhibitory, cytotoxic, and anti-migratory effects. It can reduce the frequency of multipotent/self-renewing cells in vitro and the malignant potential of primary human glioblastoma cells in vivo [70]. Therefore, niclosamide, an anthelmintic drug used to treat tapeworm infections, has recently renewed interest in its use as a treatment for diseases such as colon cancer and glioblastoma. A variety of studies have uncovered that niclosamide had effects on cell inhibition, cytotoxicity, and anti-migration and inhibited intracellular NOTCH-, mTOR-, WNT/CTNNB1-, and NF-kB signal transduction [70–73]. Among them, Oh et al. discovered that the combination of temozolomide and niclosamide could effectively reduce the metastasis and aggressiveness of GBM tumorspheres [72]. The last drug, ziritaxestat, is in phase 3 clinical trials for the treatment of idiopathic pulmonary fibrosis (IPF) but has been reported to act as an autotaxin inhibitor for cancer treatment when combined in different ways with other small compounds [74].

As multiple TCGA and GEO datasets were included, tumor samples were not evaluated for GBM phenotypes, which may have influenced the expression profiles and prognosis of GBM patients. In addition, all survival analyses were based on GBM samples only and were performed using online databases with an automatic assessment of confounding factors. And the primary screening measure in this study was prognostic value, which may have resulted in the omission of potentially valuable information. In the part of meta-analysis, most of the included studies were from China and there may be potential publication bias. Currently, drugs and genes, including the constructed ceRNA network we have identified, rely heavily on bioinformatics tools and databases. Their accuracy still needs to be validated by basic experiments and clinical trials. Therefore, in this study, we adopted and integrated multiple databases and methods for all predictions to improve the fidelity of the data.

## Conclusions

The role of ceRNAs in the progression of GBM has been reported, but only a few studies have focused on the part of ceRNAs in the prognosis of GBM patients. Furthermore, the majority of previous studies have constructed ceRNA networks based on sequential patterns of lncRNA–miRNA–mRNA in GBM. This study was one of the few to establish a GBM-related ceRNA network via mRNA–miRNA-circRNA sequential patterns. In conclusion, the findings of this study may provide new insights and potential therapeutic strategies for glioblastoma.

## Supplementary Information

**Additional file 1**. The supplementary tables of this study.

**Additional file 2**. The supplementary figures of this study.

**Additional file 3**. Some of the scripts used in this study.

**Additional file 4**. The statistical characteristics of DEmRNAs identified in this study.

**Additional file 5**. The P-values and log2 FC of DEmiRNAs identified in this study.

**Additional file 6**. The specific information of DEcircRNAs identified in this study.

**Additional file 7**. The statistical characteristics of DEcircRNAs in this study.

## Data Availability

The author confirms that data from the public database for this study have explained the source of the data in detail in the manuscript. The data can be found at the GEO data repository (https://www.ncbi.nlm.nih.gov/geo/) and TCGA data repository (http://cancergenome.nih.gov/), including the accession numbers: GSE165926, GSE103229, and GSE90886. And we have provided the related scripts used in this study and information of DEGs, including DEmRNAs, DEmiRNAs, and DEcircRNAs (Additional files 3, 4, 5, 6, 7).

## Abbreviations

DEGs: Differentially expressed genes
circRNAs: Circular RNAs
miRNAs: Micro RNAs
mRNAs: Massager RNAs
ceRNA: Competitive endogenous RNA
GO: Gene ontology
KEGG: Kyoto encyclopedia of genes and genome
K–M: Kaplan–Meier
CI: Confidence interval
HR: Hazard ratio
