# Supplementary material for: FZD1/KLF10-hsa-miR-4762-5p/miR-224-3p-circular RNAs axis as prognostic biomarkers and therapeutic targets for glioblastoma: a comprehensive report
Source: BMC Med Genomics. 2023 Feb 8;16:21. doi: 10.1186/s12920-023-01450-w (PMC9909915; doi:10.1186/s12920-023-01450-w)
Supplement: Supplementary file 1 — Additional file 1. The supplementary tables of this study. [file 12920_2023_1450_MOESM1_ESM.docx]

Supplementary Table 1. The criteria of literature inclusion and exclusion.

| Selection criteria |
| --- |
| Inclusion: |
| (1) Topic: cancer |
| (2) Diagnosis approach: histology or pathology |
| (3) Detection method: ISH, RT-qPCR, or other ways in tissues |
| (4) Associations between expression of FZD1 and KLF10 and the prognosis of cancer |
| (5) 95% CI and HR: adequate data to estimate |
| Exclusion:  (1) Type of Literature: letters, case reports, reviews and expert opinions etc.  (2) studies unrelated to FZD1 and KLF10  (3) Studies with no available data on patient outcomes |

Abbreviations: ISH in situ hybridization, RT-qPCR real time quantitative polymerase chain reaction, CI confidence interval, HR hazard ratio.

Supplementary Table 2. Characteristics of the incorporated FZD1-related studies.

| Study (year) | Country | Samples | Type | Survival analysis | Method | Cut-off | Record method | HR (95%CI) | NOS |
| --- | --- | --- | --- | --- | --- | --- | --- | --- | --- |
| Peng (2019) | China | 530 | Clear Cell Renal Cell Carcinoma | Multivariate | qRT-PCR | Median | Directly in paper | 0.569 (0.406-0.797) | 7 |
| Li (2014) | China | 80 | Gallbladder carcinoma | Multivariate | ISH | Median | Directly in paper | 2.588 (1.327-5.050) | 8 |
| Yang (2018) | China | 106 | Pancreatic Ductal Adenocarcinoma | Multivariate | ISH | Median | Directly in paper | 2.866 (1.194- 6.879) | 7 |
| Chen (2021) | China | 62 | Clear Cell Renal Cell Carcinoma | Univariate | qRT-PCR | Mean | K-M curves | 1.836 (1.035-3.255) | 7 |
| Li (2021) | China | 42 | Pancreatic adenocarcinoma | Multivariate | qRT-PCR | Mean | K-M curves | 0.7 (0.45-1.08) | 7 |
| Su (2016) | China | 152 | non-small cell lung cancer | Multivariate | ISH | Median | Directly in paper | 2.7 (1.31-5.58 | 8 |

Abbreviations: qRT-PCR quantitative reverse transcription polymerase chain reaction, ISH in situ hybridization, NOS Newcastle–Ottawa Scale, K-M curves Kaplan-Meier curves, HR hazard ratio, CI confidence interval.

Supplementary Table 3. Characteristics of the included KLF-10-related studies.

| Study (year) | Country | Samples | Type | Survival analysis | Method | Cut-off | Record method | HR (95%CI) | NOS |
| --- | --- | --- | --- | --- | --- | --- | --- | --- | --- |
| Lin (2022) | China | 121 | Gastric Cancer | Multivariate | ISH | Median | Directly in paper | 2.344 (1.460–3.762) | 7 |
| Ye (2021) | China | 286 | Oral Squamous Cell Carcinoma | Multivariate | ISH | Median | Directly in paper | 1.528 (1.031–2.265) | 7 |
| Chang (2012) | China | 85 | Pancreatic Adenocarcinoma | Univariate | ISH | Mean | K-M curves | 1.59 (0.32-7.53) | 8 |
| Zhao (2022) | China | 48 | melanoma | Univariate | qRT-PCR | Mean | K-M curves | 2.701 (1.304-5.587) | 7 |
| Ferraro (2005) | USA | 125 | non-small cell lung cancer | Univariate | qRT-PCR | Mean | K-M curves | 4.16 (1.52-7.84) | 8 |

Abbreviations: qRT-PCR quantitative reverse transcription polymerase chain reaction, ISH in situ hybridization, NOS Newcastle–Ottawa Scale, K-M curves Kaplan-Meier curves, HR hazard ratio, CI confidence interval.

Supplementary Table 4. Subgroup analysis of FZD1 for OS in human cancers.

| Variables | Studies | Samples | Model | HR (95% CI) | I^2^ (%) | χ^2^ | | p-value | |  |
| --- | --- | --- | --- | --- | --- | --- | --- | --- | --- | --- |
| OS | 6 | 972 | Fixed | 2.55 (1.95-3.34) | 39 | 8.19 | | 0.15 | |  |
| Record method |  |  |  |  |  |  | |  | |  |
| Directly | 4 | 868 | Random | 2.66 (2.00-3.55) | 59 | 7.37 | | 0.06 | |  |
| K-M curves | 2 | 104 | Fixed | 1.85 (0.85-4.05) | 0 | 0.1 | | 0.75 | |  |
| Method |  |  |  |  |  |  | |  | |  |
| ISH | 3 | 338 | Fixed | 1.86 (1.20-2.88) | 31 | 2.91 | | 0.23 | |  |
| qRT-PCR | 3 | 634 | Fixed | 3.09 (2.19-4.35) | 6 | | 2.13 | | 0.34 | |
| Cancer Type |  |  |  |  |  | |  | |  | |
| Digestive System | 3 | 228 | Fixed | 2.51 (1.47-4.31) | 0 | | 0.32 | | 0.85 | |
| Urinary System | 2 | 592 | Fixed | 3.18 (2.22-4.55) | 44 | | 1.79 | | 0.18 | |
| Respiratory System | 1 | 152 | - | 2.7 (1.31-5.58 | - | | - | | - | |

Abbreviations: qRT-PCR quantitative reverse transcription polymerase chain reaction, ISH in situ hybridization, HR hazard ratio, 95% CI 95% confidence interval.

Supplementary Table 5. Subgroup analysis of KLF10 for OS in human cancers.

| Variables | Studies | Samples | Model | HR (95% CI) | I^2^ (%) | χ^2^ | p-value |
| --- | --- | --- | --- | --- | --- | --- | --- |
| OS | 5 | 665 | Fixed | 1.41 (1.02-1.97) | 16 | 6.19 | 0.32 |
| Method |  |  |  |  |  |  |  |
| qRT-PCR | 2 | 173 | Random | 1.46 (0.79-2.70) | 78 | 3.46 | 0.03 |
| ISH | 3 | 492 | Fixed | 1.40 (0.94-2.07) | 11 | 0.28 | 0.87 |
| Record method |  |  |  |  |  |  |  |
| Directly | 2 | 307 | Fixed | 1.03 (0.67-1.60) | 35 | 1.53 | 0.22 |
| K-M curves | 3 | 358 | Fixed | 1.48 (0.89-2.45) | 44 | 3.60 | 0.17 |
| Cancer Type |  |  |  |  |  |  |  |
| Digestive System | 2 | 206 | Random | 1.02 (0.59-1.77) | 54 | 2.19 | 0.14 |
| Respiratory System | 1 | 125 | - | 4.16 (1.52-7.84) | - | - | - |
| Other | 2 | 334 | Fixed | 1.52 (0.91-2.54) | 62 | 2.61 | 0.11 |

Abbreviations: qRT-PCR quantitative reverse transcription polymerase chain reaction, ISH in situ hybridization, HR hazard ratio, 95% CI 95% confidence interval.
