# Supplementary material for: FZD1/KLF10-hsa-miR-4762-5p/miR-224-3p-circular RNAs axis as prognostic biomarkers and therapeutic targets for glioblastoma: a comprehensive report
Source: BMC Med Genomics. 2023 Feb 8;16:21. doi: 10.1186/s12920-023-01450-w (PMC9909915; doi:10.1186/s12920-023-01450-w)
Supplement: Supplementary file 2 — Additional file 2. The supplementary figures of this study. [file 12920_2023_1450_MOESM2_ESM.docx]

**Supplementary Figures**


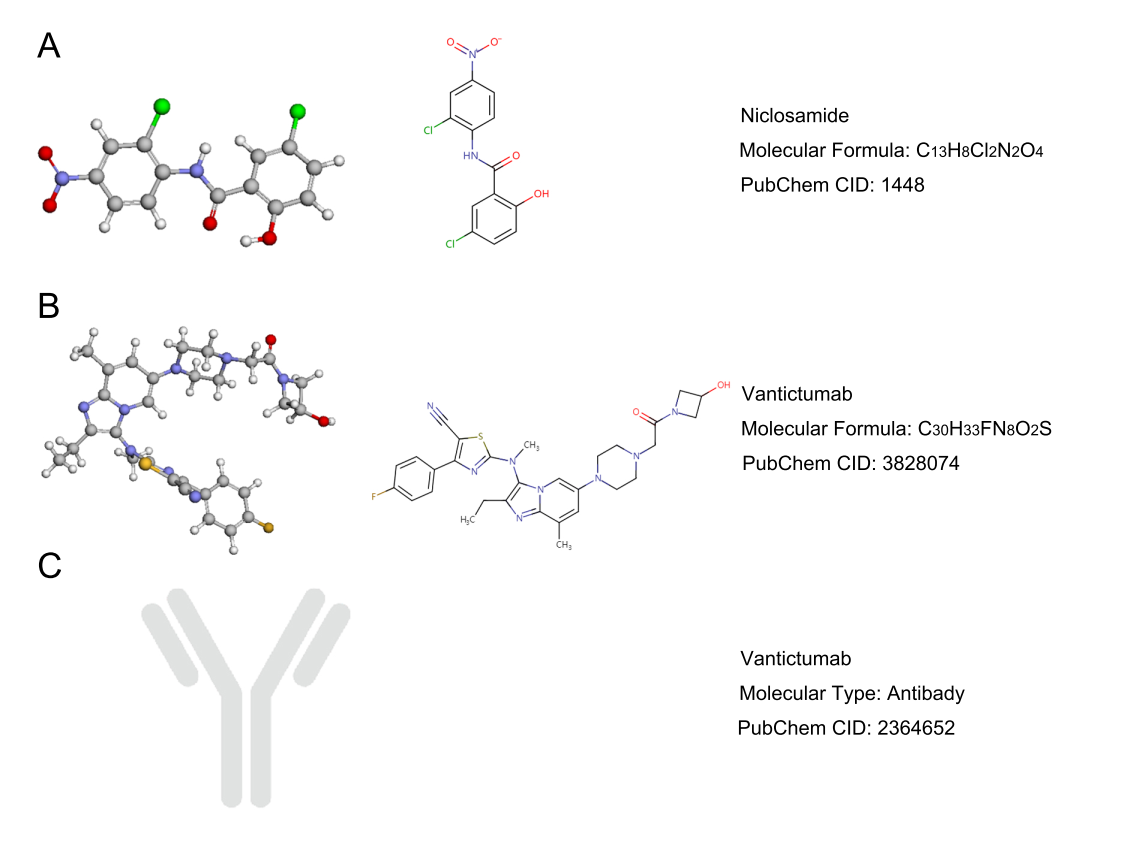


**Supplementary Figure 1.** The 2D structures of three drugs identified by the DGIdb database. A Niclosamide. B Ziritaxestat. C Vantictumab.


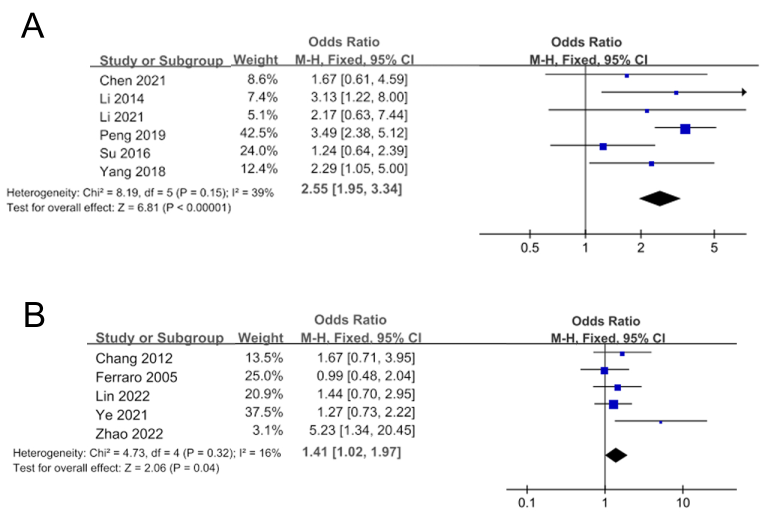


**Supplementary Figure 2.** Forest plot of the associations between FZD1 (A) and KLF10 (B) expression and overall survival.


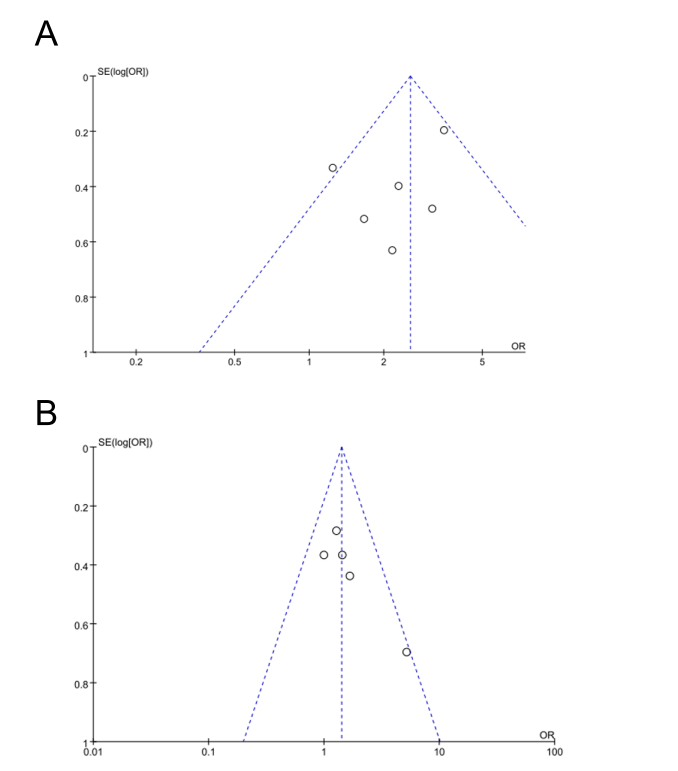


**Supplementary Figure 3.** Funnel plot of the publication bias for overall survival. A represents the FZD1-related meta-analysis and B represents the KLF10-related meta-analysis.
