# Supplementary material for: FZD1/KLF10-hsa-miR-4762-5p/miR-224-3p-circular RNAs axis as prognostic biomarkers and therapeutic targets for glioblastoma: a comprehensive report
Source: BMC Med Genomics. 2023 Feb 8;16:21. doi: 10.1186/s12920-023-01450-w (PMC9909915; doi:10.1186/s12920-023-01450-w)
Supplement: Supplementary file 3 — Additional file 3. Some of the scripts used in this study. [file 12920_2023_1450_MOESM3_ESM.doc]

#if (!requireNamespace("BiocManager", quietly = TRUE))

# install.packages("BiocManager")

#BiocManager::install("limma")

#install.packages("pheatmap")

#引用包

library(limma)

library(pheatmap)

inputFile="circMatrix2.txt" #输入文件

conFile="sample1.txt" #对照组样品

treatFile="sample2.txt" #实验组样品

logFCfilter=2 #logFC过滤阈值

adj.P.Val.Filter=0.05 #矫正后p值阈值

setwd("C:\\technique\\06.circDiff") #设置工作目录

#读取输入文件，并对输入文件整理

rt=read.table(inputFile,sep="\t",header=T,check.names=F)

rt=as.matrix(rt)

rownames(rt)=rt[,1]

exp=rt[,2:ncol(rt)]

dimnames=list(rownames(exp),colnames(exp))

data=matrix(as.numeric(as.matrix(exp)),nrow=nrow(exp),dimnames=dimnames)

data=avereps(data)

data=data[rowMeans(data)>0,]

#data=log2(data+1) #如果表达数值很大，需要对数据取log2，可以这行前面#号删掉

data=normalizeBetweenArrays(data)

#读取样品信息

sample1=read.table(conFile,sep="\t",header=F,check.names=F)

sample2=read.table(treatFile,sep="\t",header=F,check.names=F)

conData=data[,as.vector(sample1[,1])]

treatData=data[,as.vector(sample2[,1])]

data=cbind(conData,treatData)

conNum=ncol(conData)

treatNum=ncol(treatData)

#差异分析

Type=c(rep("con",conNum),rep("treat",treatNum))

design <- model.matrix(~0+factor(Type))

colnames(design) <- c("con","treat")

fit <- lmFit(data,design)

cont.matrix<-makeContrasts(treat-con,levels=design)

fit2 <- contrasts.fit(fit, cont.matrix)

fit2 <- eBayes(fit2)

allDiff=topTable(fit2,adjust='fdr',number=200000)

allDiffOut=rbind(id=colnames(allDiff),allDiff)

write.table(allDiffOut,file="circ.all.xls",sep="\t",quote=F,col.names=F)

#输出矫正后的表达量

outData=rbind(id=paste0(colnames(data),"_",Type),data)

write.table(outData,file="circ.normalize.txt",sep="\t",quote=F,col.names=F)

#输出差异结果

diffSig=allDiff[with(allDiff, (abs(logFC)>logFCfilter & adj.P.Val < adj.P.Val.Filter )), ]

diffSigOut=rbind(id=colnames(diffSig),diffSig)

write.table(diffSigOut,file="circ.diff.xls",sep="\t",quote=F,col.names=F)

write.table(diffSigOut,file="circ.diff.txt",sep="\t",quote=F,col.names=F)

#绘制差异基因热图

geneNum=50

diffSig=diffSig[order(as.numeric(as.vector(diffSig$logFC))),]

diffGeneName=as.vector(rownames(diffSig))

diffLength=length(diffGeneName)

hmGene=c()

if(diffLength>(2*geneNum)){

hmGene=diffGeneName[c(1:geneNum,(diffLength-geneNum+1):diffLength)]

}else{

hmGene=diffGeneName

}

hmExp=data[hmGene,]

Type=c(rep("C",conNum),rep("T",treatNum))

names(Type)=colnames(data)

Type=as.data.frame(Type)

pdf(file="circ.heatmap.pdf",height=10,width=8)

pheatmap(hmExp,

annotation=Type,

color = colorRampPalette(c("blue", "white", "red"))(50),

cluster_cols =F,

show_colnames = F,

scale="row",

fontsize = 10,

fontsize_row=8,

fontsize_col=10)

dev.off()

#install.packages("venn")

library(venn) #引用包

outFile="intersect.miRNA.txt" #输出文件名称

setwd("C:\\technique\\14.mirnaVenn") #设置工作目录

geneList=list()

#读取差异miRNA

rt=read.table("mirna.diff.txt",sep="\t",header=T,check.names=F)

geneNames=as.vector(rt[,1]) #提取miRNA名称

geneNames=gsub("^ | $","",geneNames) #去掉miRNA首尾的空格

uniqGene=unique(geneNames) #miRNA取unique

geneList[["diff miRNA"]]=uniqGene

#读取MRE

rt=read.table("MRE.txt",sep="\t",header=T,check.names=F)

geneNames=as.vector(rt[,2]) #提取miRNA名称

geneNames=gsub("^ | $","",geneNames) #去掉miRNA首尾的空格

uniqGene=unique(geneNames) #miRNA取unique

geneList[["MRE"]]=uniqGene

#绘制venn图

mycol=c("#029149","#E0367A","#5D90BA","#431A3D","#FFD121","#D8D155","#223D6C","#D20A13","#088247","#11AA4D","#7A142C","#5D90BA","#64495D","#7CC767")

pdf(file="miRNA.venn.pdf",width=5,height=5)

venn(geneList,col=mycol[1:length(geneList)],zcolor=mycol[1:length(geneList)],box=F,ilabels=F)

dev.off()

#保存交集miRNA

intersectGenes=Reduce(intersect,geneList)

write.table(file=outFile,intersectGenes,sep="\t",quote=F,col.names=F,row.names=F)

#install.packages("venn")

library(venn) #引用包

outFile="intersect.mRNA.txt" #输出文件名称

setwd("C:\\technique\\16.mrnaVenn") #设置工作目录

geneList=list()

#读取差异mRNA

rt=read.table("mrna.diff.txt",sep="\t",header=T,check.names=F)

geneNames=as.vector(rt[,1]) #提取基因名称

geneNames=gsub("^ | $","",geneNames) #去掉基因首尾的空格

uniqGene=unique(geneNames) #基因取unique

geneList[["diff mRNA"]]=uniqGene

#读取靶基因

rt=read.table("target.txt",sep="\t",header=T,check.names=F)

geneNames=as.vector(rt[,2]) #提取靶基因名称

geneNames=gsub("^ | $","",geneNames) #去掉靶基因首尾的空格

uniqGene=unique(geneNames) #靶基因A取unique

geneList[["miRNA target"]]=uniqGene

#绘制venn图

mycol=c("#029149","#E0367A","#5D90BA","#431A3D","#FFD121","#D8D155","#223D6C","#D20A13","#088247","#11AA4D","#7A142C","#5D90BA","#64495D","#7CC767")

pdf(file="mRNA.venn.pdf",width=5,height=5)

venn(geneList,col=mycol[1:length(geneList)],zcolor=mycol[1:length(geneList)],box=F,ilabels=F)

dev.off()

#保存交集基因

intersectGenes=Reduce(intersect,geneList)

write.table(file=outFile,intersectGenes,sep="\t",quote=F,col.names=F,row.names=F)

#install.packages("ggpubr")

#引用包

library(ggpubr)

library(reshape2)

setwd("C:\\technique\\20.ceRNAboxplot") #设置工作目录

#定义boxplot函数

bioBoxplot=function(expFile=null,geneFile=null,diffFile=null,outFile=null,height=null,width=null){

#读取输入文件，并对输入文件整理

rt=read.table(expFile,sep="\t",header=T,check.names=F,row.names=1)

gene=read.table(geneFile,sep="\t",header=F,check.names=F)

data=rt[as.vector(gene[,1]),]

Type=gsub("(.*?)\\_(.*)","\\2",colnames(data))

Type=ifelse(Type=="con","C","T")

colnames(data)=gsub("(.*?)\\_(.*)","\\1",colnames(data))

#读取差异文件

diff=read.table(diffFile,sep="\t",header=T,check.names=F,row.names=1)

diff=diff[as.vector(gene[,1]),]

adjPval=as.character(diff$adj.P.Val)

Sig=ifelse(adjPval<0.001,"***",ifelse(adjPval<0.01,"**",ifelse(adjPval<0.05,"*","")))

data=t(data)

data=cbind(as.data.frame(data),Type)

data=melt(data,id.vars=c("Type"))

colnames(data)=c("Type","Gene","Expression")

yMax=max(data$Expression) #定义显著性的y坐标值

p=ggboxplot(data, x="Gene", y="Expression", color = "Type", orientation = "horizontal",

ylab="", add = "none", xlab="",width=0.8,

palette = c("blue","red"))+rotate_x_text(60)+

geom_text(data=diff,aes(label=Sig,x=row.names(diff), y=yMax), position=position_dodge(1), vjust=0)

pdf(file=outFile,width=width,height=height) #输出图片文件

print(p)

dev.off()

}

#绘制circRNA箱线图

bioBoxplot(expFile="circ.normalize.txt",

geneFile="ceRNA.circList.txt",

diffFile="circ.diff.txt",

outFile="circ.boxplot.pdf",

height=5, width=5)

#绘制miRNA箱线图

bioBoxplot(expFile="mirna.normalize.txt",

geneFile="ceRNA.mirnaList.txt",

diffFile="mirna.diff.txt",

outFile="mirna.boxplot.pdf",

height=4, width=5)

#绘制mRNA箱线图

bioBoxplot(expFile="mrna.normalize.txt",

geneFile="ceRNA.mrnaList.txt",

diffFile="mrna.diff.txt",

outFile="mrna.boxplot.pdf",

height=5, width=5)

#if (!requireNamespace("BiocManager", quietly = TRUE))

# install.packages("BiocManager")

#BiocManager::install("limma")

library(limma) #引用包

expFile="symbol.txt" #表达数据文件

cliFile="time.txt" #临床数据文件

geneFile="ceRNA.mrnaList.txt" #基因列表文件

setwd("C:\\technique\\28.mergeTime") #工作目录（需修改）

#读取表达文件，并对输入文件整理

rt=read.table(expFile,sep="\t",header=T,check.names=F)

rt=as.matrix(rt)

rownames(rt)=rt[,1]

exp=rt[,2:ncol(rt)]

dimnames=list(rownames(exp),colnames(exp))

data=matrix(as.numeric(as.matrix(exp)),nrow=nrow(exp),dimnames=dimnames)

data=avereps(data)

data=data[rowMeans(data)>0,]

#读取基因列表

gene=read.table(geneFile,header=F,sep="\t",check.names=F)

sameGene=intersect(as.vector(gene[,1]),row.names(data))

data=data[sameGene,]

#删掉正常样品

group=sapply(strsplit(colnames(data),"\\-"),"[",4)

group=sapply(strsplit(group,""),"[",1)

group=gsub("2","1",group)

data=data[,group==0]

colnames(data)=gsub("(.*?)\\-(.*?)\\-(.*?)\\-(.*?)\\-.*","\\1\\-\\2\\-\\3",colnames(data))

data=t(data)

data=avereps(data)

#读取生存数据

cli=read.table(cliFile,sep="\t",check.names=F,header=T,row.names=1) #读取临床文件

#数据合并并输出结果

sameSample=intersect(row.names(data),row.names(cli))

data=data[sameSample,]

cli=cli[sameSample,]

out=cbind(cli,data)

out=cbind(id=row.names(out),out)

write.table(out,file="expTime.txt",sep="\t",row.names=F,quote=F)

#install.packages("survival")

#install.packages("survminer")

#引用包

library(survival)

library(survminer)

inputFile="expTime.txt" #输入文件

col=c("red","blue") #定义高低表达组的颜色

setwd("C:\\technique\\29.survival") #设置工作目录

rt=read.table(inputFile,header=T,sep="\t",check.names=F,row.names=1) #读取输入文件

rt$futime=rt$futime/365 #生存单位改成年

#对基因进行循环

outTab=data.frame()

for(gene in colnames(rt)[3:ncol(rt)]){

group=ifelse(rt[,gene]>median(rt[,gene]),"high","low")

diff=survdiff(Surv(futime, fustat) ~group,data = rt)

pValue=1-pchisq(diff$chisq,df=1)

if(pValue<0.05){

outVector=cbind(gene,pValue)

outTab=rbind(outTab,outVector)

if(pValue<0.001){

pValue="p<0.001"

}else{

pValue=paste0("p=",sprintf("%.03f",pValue))

}

fit <- survfit(Surv(futime, fustat) ~ group, data = rt)

#绘制生存曲线

surPlot=ggsurvplot(fit,

data=rt,

pval=pValue,

pval.size=6,

legend.labs=c("high","low"),

legend.title=paste0(gene," levels"),

font.legend=12,

xlab="Time(years)",

palette=col,

break.time.by = 2,

conf.int=T,

fontsize=5,

risk.table=TRUE,

ylab="Overall survival",

risk.table.title="",

risk.table.height=.25)

pdf(file=paste0(gene,".pdf"),onefile = FALSE,

width = 6, #图片的宽度

height =5) #图片的高度

print(surPlot)

dev.off()

}

}

#输出基因和p值表格文件

write.table(outTab,file="survival.result.txt",sep="\t",row.names=F,quote=F)
